# Gluteal silicone injections and total hip arthroplasty: a case report

**DOI:** 10.1186/1752-1947-8-140

**Published:** 2014-05-06

**Authors:** Jörn B Seeger, Gafar A Ahmed, Erhan Basad, Markus Rickert, Bernd A Ishaque

**Affiliations:** 1 Department of Orthopaedics and Orthopaedic Surgery, University Hospital Giessen and Marburg (UKGM), Klinikstrasse 33, 35392 Giessen, Germany; 2 ATOS Klinik Heidelberg, Bismarckstrasse 9-15, 69115 Heidelberg, Germany

**Keywords:** Silicone, Injections, Avascular necrosis, Granuloma, Hip replacement

## Abstract

**Introduction:**

Silicone injection is a common procedure in cosmetic surgery. Granuloma formation and migration are the most commonly observed complications.

**Case presentation:**

We report an unusual case of avascular necrosis of the hip in a 41-year-old woman from Thailand presenting with hip pain. Subcutaneous nodules were observed in the clinical examination. A pelvic X-ray revealed necrosis of the right femoral head and histological analysis of the punctuated nodules showed a reaction of foreign body granulomas. During surgical treatment with a hip replacement solitary silicone cysts were removed.

**Conclusions:**

This case report emphasizes that orthopedic surgeons treating patients with necrosis of the hip joint in combination with palpable granulomas in the gluteal region have to be aware of silicone augmentation and its potential complications before planning a hip replacement.

## Introduction

Although adverse pulmonary reactions, including diffuse alveolar damage, pneumonitis, and acute adult respiratory distress syndrome (ARDS) have been described as a result of silicone injections [1-3], silicone is often applied in cosmetic procedures. Granuloma formation and migration of injected silicone present the most common complications [4,5].

Nevertheless, intramuscular gluteoplasty augmentation with gluteal implants offers the best predictable long-term results with only a low incidence of major complications [6].

## Case presentation

In May 2012, a 41-year old Asian woman from Thailand presented with consistent pain of the right hip joint since December 2011. She reported a polytrauma with fractures of the zygomatic bone in 1990 in Thailand. After the polytrauma, our patient was treated in an intensive care unit in Thailand for a couple of weeks. After remission, a nose reconstruction was performed with silicone in 1991. In addition our patient suffered from fibromyalgia and osteoporosis. Due to the pain in her paranasal sinus, prolonged cortisone therapy was carried out.

A clinical examination revealed a reduced range of motion (ROM) with pain beginning in the right thigh at a flexion of 20°. Full weight bearing was possible, but our patient presented an insecure walking gait characterized by small steps. As a secondary finding, multiple firm, tender subcutaneous nodules with a dense aspect could be observed as well as cicatrices in the gluteal region.

**Figure 1:**
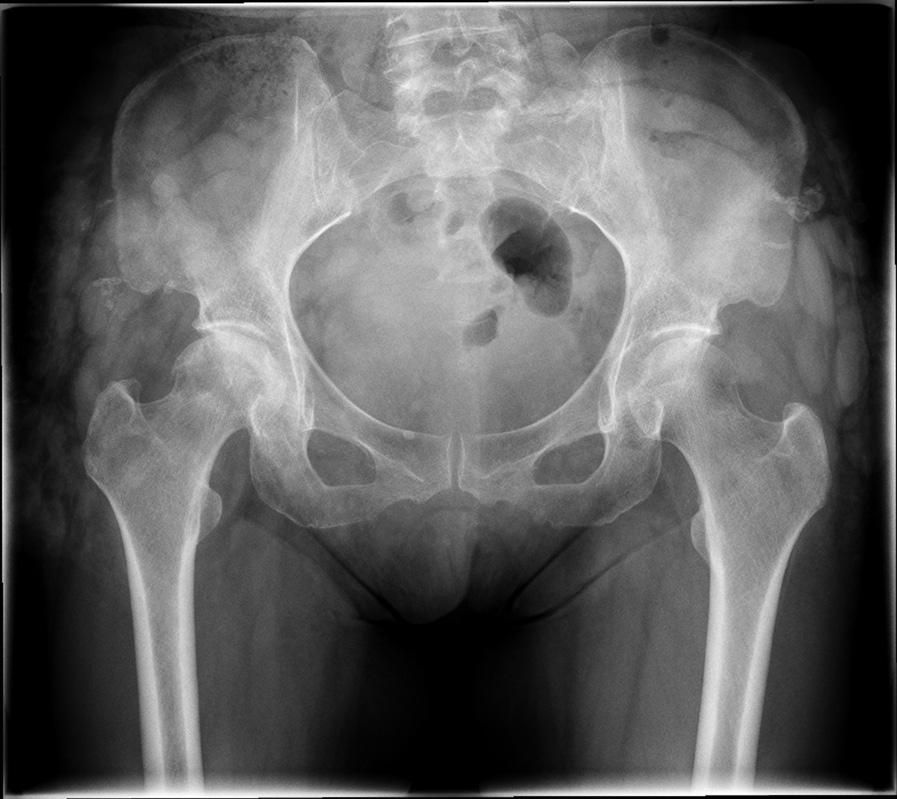
Pelvic X-ray.

Pelvic X-ray showed osteoarthritis of the hip joint on both sides with osteolysis in terms of necrosis of the right femoral head (Figure 1). In addition, unclear radiopaque shadows were presumed around the pelvis.

The magnetic resonance imaging (MRI) scans performed showed a bilateral necrosis of the femoral head as well as heterotopic ossifications justifying the indication of total hip replacement. Dual-energy X-ray absorptiometry (DEXA) measurements proved osteoporosis.

Blood test results in terms of hepatitis B and C, dengue fever as well as human immunodeficiency virus (HIV) 1 and 2 were negative so that a possible infection could be excluded.

**Figure 2:**
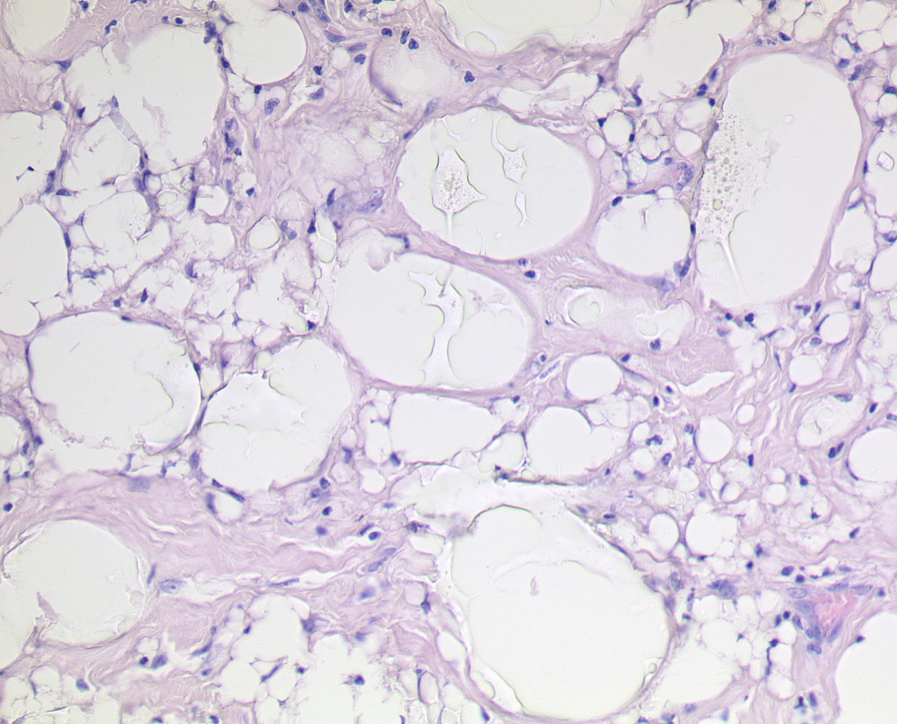
Histopathologic analysis of silicone granuloma (magnification, hematoxylin and eosin stain).

The findings in the soft tissue were sonographically punctuated and jellylike hyaline spots were described by the radiologist. The histological analysis proved a reaction of foreign body granulomas (Figure 2). There was no evidence for malignancy.

A few days before the hip replacement, our patient admitted having had silicone injections in the gluteal region approximately 15 years ago.

**Figure 3:**
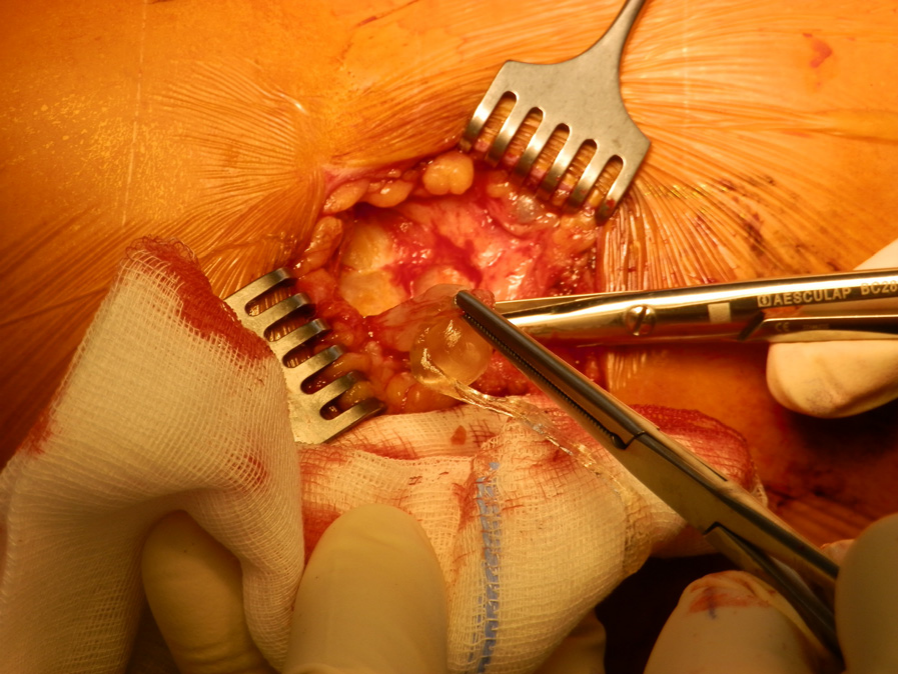
Intraoperative resection of silicone cysts.

**Figure 4:**
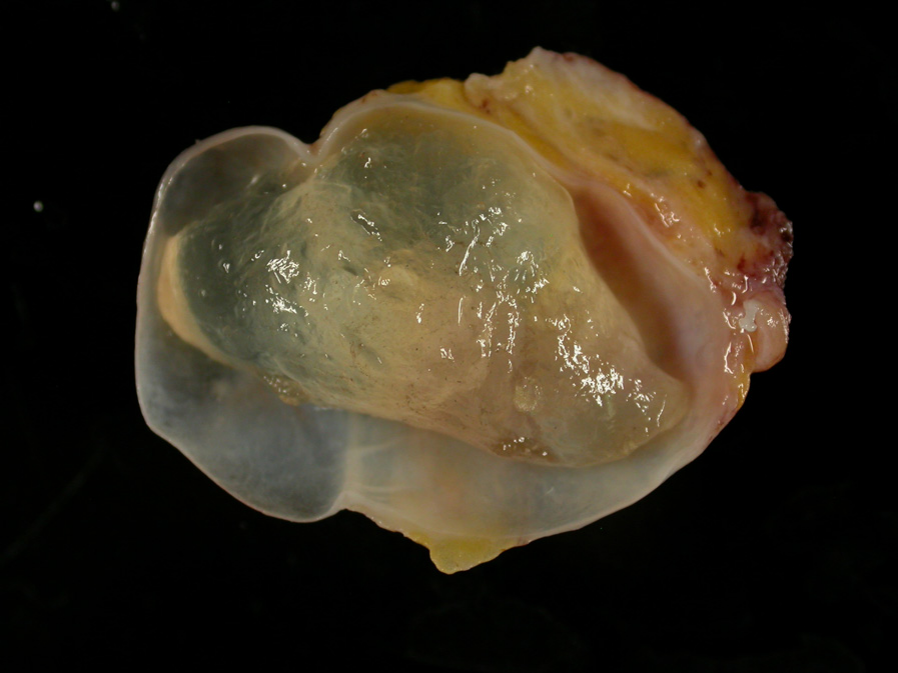
Macroscopic picture of resected silicone cyst.

**Figure 5:**
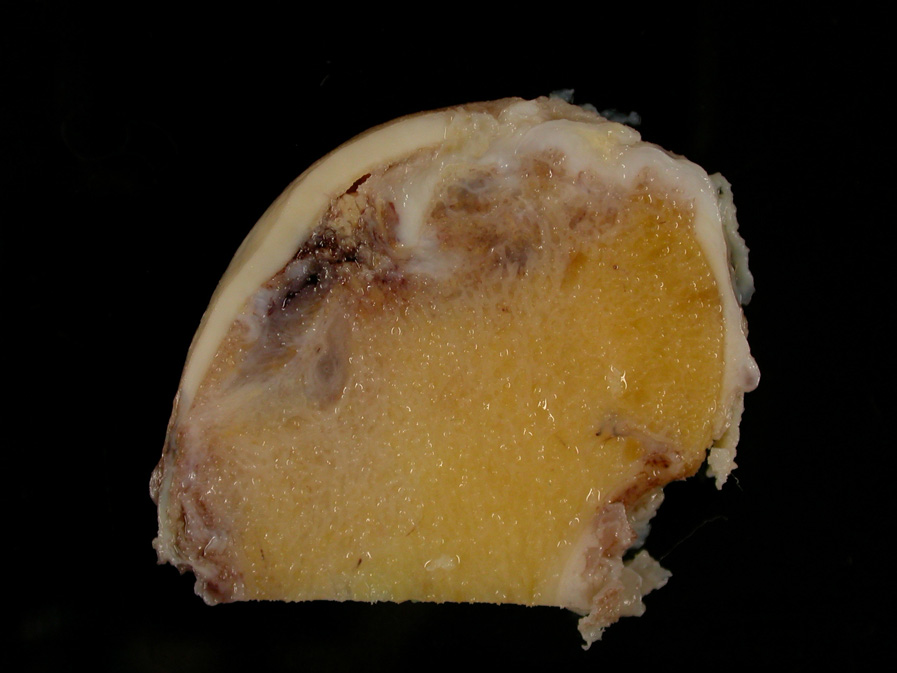
Macroscopic picture of the femoral head with necrosis.

Our patient was given understandable and detailed information about an increased infection risk during her hip replacement due to the silicone granulomas. During the surgical procedure for hip replacement her highly scarred subcutis was prepared and solitary silicone cysts as well as granulomas were removed (Figure 3). The content of these cysts appeared ‘like glue’ as seen in Figure 4. The femoral head showed advanced necrotic areas (Figure 5). After surgery, our patient improved and wound healing occurred without any complications so our patient was discharged from hospital. The microbiologic analysis described no pathogenic infection after 14 days.

On follow-up examination our patient’s ROM had improved significantly and she reported no pain. Due to her excellent postoperative results a second hip replacement, on the left side, is planned shortly.

## Discussion

The first cases of gluteal augmentation in the literature were described by Bartels using a mammary prothesis in the gluteal region [7]. Vergara and Amezcua reported on 15 years’ experience with intramuscular gluteal implants showing the best predictable long-term results with no irregularities [6].

Silicone granulomas after soft-tissue augmentation of the gluteal region may occur years to decades after injection. The first occurrence of silicone granuloma was reported by Winer in 1964 [8]. Granulomas following free silicone gluteal augmentation have also been described by Wosnitzer [9].

In our case, lucencies on X-ray imaging of the pelvis initially were assumed to reflect heterotopic ossifications. The long-term intensive care over several weeks after polytrauma might have had an influence on the formation of these ossifications.

After recovery, our patient received multiple silicone injections in the gluteal region in the further course. Years later, our patient presented with avascular necrosis of the right femoral head. The cause for the necrosis remains uncertain but the prolonged cortisone therapy also has to be considered.

The patient admitted having had silicone injections in the past. When observing the palpatory changes of the soft tissue in the gluteal region, the lucencies seen on X-ray imaging emphasized the possibility of being silicone granulomas. In the further course, these granulomas were explored preoperatively with sonographic punctuation. Microbiologic analyses showed no indication for infection so the hip replacement was carried out.

According to Ellis *et al*. liquid silicone will continue to be misused by untrained injectors and complications will continue to occur [10]. Serious complications such as severe edema of the area injected, localized discoloration of the area injected as well as cellulitis, ulcerations, migration and nodule formation have been reported with silicone injections [11,12]. Silicone injection has been described as leading to multisystem organ failure [13]. Vergara *et al*. recommend intramuscular gluteoplasty augmentation with gluteal implants as offering the best results with a low incidence of major complications [6].

Patients often do not divulge a history of silicone augmentation [4]. Orthopedic surgeons treating patients with necrosis of the hip joint in combination with palpable granulomas in the gluteal region have to be aware of silicone augmentation and its potential complications before planning a hip replacement.

## Conclusions

This case report emphasizes that orthopedic surgeons treating patients with necrosis of the hip joint in combination with palpable granulomas in the gluteal region have to be aware of silicone augmentation and its potential complications before planning a hip replacement.

## Consent

Written informed consent was obtained from the patient for publication of this case report and any accompanying images. A copy of the written consent is available for review by the Editor-in-Chief of this journal.

## Abbreviations

ARDS: Acute respiratory distress syndrome; DEXA: dual-energy X-ray absorptiometry; MRI: magnetic resonance imaging; ROM: range of motion.

## Competing interests

The authors declare that they have no competing interests.

## Authors’ contributions

JBS carried out the patient’s study, participated in collecting the data and drafted the manuscript. GAA, EB and MR participated in the design of the study and helped to draft the manuscript. BAI conceived the study and participated in the design and coordination. All authors read and approved the final manuscript.
